# Probing Majorana bound states via counting statistics of a single electron transistor

**DOI:** 10.1038/srep11416

**Published:** 2015-06-22

**Authors:** Zeng-Zhao Li, Chi-Hang Lam, J. Q. You

**Affiliations:** 1 Laboratory for Quantum Optics and Quantum Information, Beijing Computational Science Research Center, Beijing 100094, China; 2 Department of Applied Physics, Hong Kong Polytechnic University, Hung Hom, Hong Kong, China

## Abstract

We propose an approach for probing Majorana bound states (MBSs) in a nanowire via counting statistics of a nearby charge detector in the form of a single-electron transistor (SET). We consider the impacts on the counting statistics by both the local coupling between the detector and an adjacent MBS at one end of a nanowire and the nonlocal coupling to the MBS at the other end. We show that the Fano factor and the skewness of the SET current are minimized for a symmetric SET configuration in the absence of the MBSs or when coupled to a fermionic state. However, the minimum points of operation are shifted appreciably in the presence of the MBSs to asymmetric SET configurations with a higher tunnel rate at the drain than at the source. This feature persists even when varying the nonlocal coupling and the pairing energy between the two MBSs. We expect that these MBS-induced shifts can be measured experimentally with available technologies and can serve as important signatures of the MBSs.

Majorana fermions are particles that are their own antiparticles. In high-energy physics, neutrino being an elementary particle was suggested as a Majorana fermion^1^. Experiments aiming to prove this proposal are still on going. Besides the high-energy context where they arose, it is believed that Majorana fermions can also emerge as quasiparticles in condensed-matter systems^2^,^3^. The search for Majorana bound states (MBSs) in these systems has attracted much interest not only due to their exotic properties (e.g., non-Abelian statistics) but also because they are promising candidates for topological quantum computation^4^,^5^. Several physical systems have been suggested to support MBSs, including fractional quantum Hall states^6^,^7^,^8^, chiral *p*-wave superconductors/superfluids^8^,^9^ surfaces of three-dimensional (3D) topological insulators in proximity to an *s*-wave superconductor^10^, superfluids in the 3He-B phase^11^,^12^, and helical edge modes of 2D topological insulators in proximity to both a ferromagnet and a superconductor^13^. More recently, it has been shown that a spin-orbit coupled semiconducting 2D thin film^14^ or a 1D nanowire^15^,^16^,^17^,^18^,^19^ with Zeeman spin splitting, which is in proximity to an *s*-wave superconductor, can also host MBSs.

Providing experimental evidences for the realization of MBSs is of great importance. Techniques proposed to detect MBSs include the analysis of the tunneling spectroscopy^20^,^21^,^22^,^23^, the verification of the nature of nonlocality^13^,^24^ or the observation of the periodic Majorana-Josephson current^25^. In particular, the very recent observation of a zero-bias peak in the differential conductance through a semiconductor nanowire in contact with a superconducting electrode indicated the possible existence of a midgap Majorana state^26^. Such a zero-bias peak was also observed in subsequent experiments^27^,^28^,^29^. However, this zero-bias peak could be due to the Kondo resonance^30^ and also occur in the presence of either disorders^31^ or a singlet-doublet quantum phase transition^32^, corresponding to ordinary Andreev bound states rather than MBSs. Moreover, a study of a more realistic model of a nanowire with MBSs further indicates a different origin for this observed zero-bias peak^33^. There are several recent works^34^,^35^,^36^,^37^,^38^,^39^ developed, for example, to distinguish between the Majorana and Kondo origins of the zero-bias conductance peak, but a definite evidence for the zero-bias anomaly due to MBSs is still missing. Therefore, further investigations are needed to convincingly reveal the existence of MBSs.

We will focus on the detection of MBSs which exist in pairs at the two ends of a nanowire. Most previous studies based on a variety of setups considered a detector coupled locally to an adjacent MBS at one end of the nanowire only^13^,^21^,^40^,^41^,^42^, as the coupling to the other MBS farther away is neglected. For example, a quantum dot coupled to a MBS was studied in Ref. 40. The current and the shot noise through the quantum dot were calculated. A characteristic feature in the frequency dependence of the shot noise was proposed as a signature for the MBS. The coupling of a quantum dot to two MBSs at both ends of a nanowire has also been studied^21^, but only the conductance was reported. In this work, we study both the local and nonlocal coupling of a single electron transistor (SET) (consisting of a quantum dot and two electrodes) to two MBSs at both ends of a nanowire. We calculate the full counting statistics (FCS)^43^,^44^ of electron transport through the SET. FCS yields all zero-frequency current correlations at once and provides detailed insights into the nature of charge transfer beyond what is available from conductance measurements alone^45^,^46^. Importantly, it has also become an experimentally accessible technique in recent years^47^,^48^,^49^. Using the FCS, we calculate the current, Fano factor and skewness as functions of a tunnel rate ratio of the SET. The calculations are performed for various couplings of the SET island with the MBSs. The results are also compared with those for coupling to a fermionic state instead. We will show in the following that in the absence of the MBSs or when coupled to fermionic states, the Fano factor and the skewness are minimized for a symmetric SET. However, in the presence of the MBSs, the minimum points shift appreciably to occur for an asymmetric SET with a higher tunnel rate at the drain than at the source. We propose that these MBS-induced shifts of the minimum points of the Fano factor and the skewness can be used as signatures for the identification of the MBSs.

## Results

**Figure 1:**
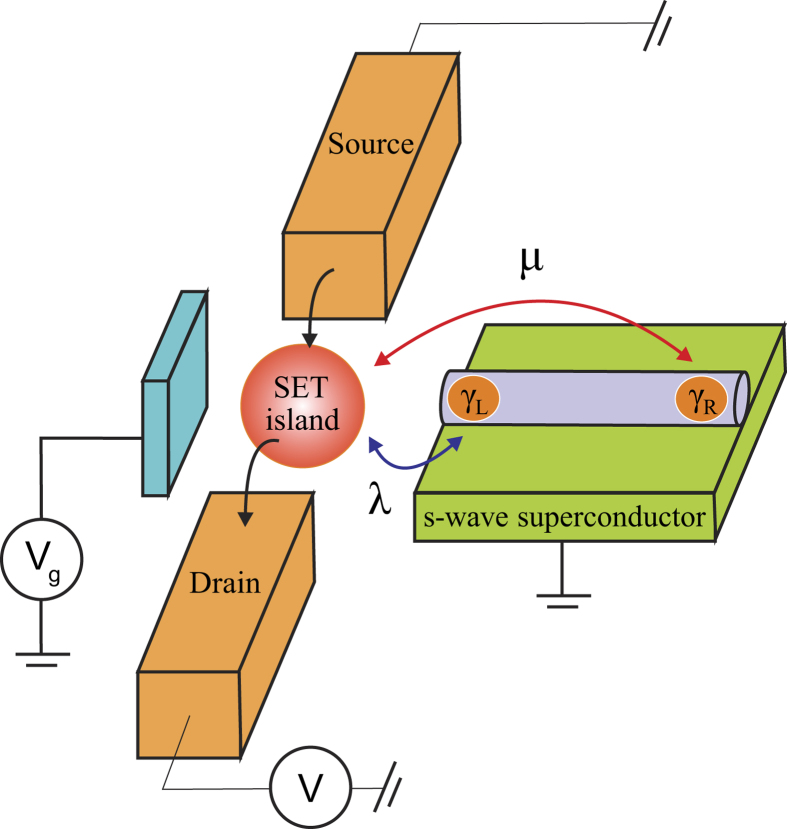
The coupled MBS-SET system. Schematic diagram of the hybrid quantum system consisting of two MBSs and a SET. The MBSs locate at the two ends of a nanowire with large Zeeman splitting and strong spin-orbit coupling, which is in proximity to an *s*-wave superconductor. The SET island is coupled to the source and drain electrodes via tunneling barriers and capacitively biased by an external gate voltage *V*_*g*_. The energy level of the SET island is tuned to be zero, i.e., in resonance with the MBSs. Also, the SET island couples to the adjacent MBS with a coupling strength *λ* and the MBS at the other end of the nanowire with a coupling strength *μ*.

The hybrid system consists of two MBSs and a SET as schematically shown in Fig. 1. With a conventional *s*-wave superconductor and a modest magnetic field, the MBSs as electron-hole quasiparticle excitations have been suggested to exist at the two ends of a semiconductor nanowire with strong spin-orbit coupling^14^,^18^,^19^. The SET consists of a metallic island coupled via tunneling barriers to two electrodes. The energy levels and the tunneling barriers can be tuned by the gate voltages. By assuming a Zeeman splitting much larger than the MBS-SET coupling strength, the source-drain bias voltage across the SET, and the tunneling rates with the source and drain electrodes, the SET island can be modeled by a single resonant level occupied by a spin-polarized electron.

The interaction between the MBSs and the SET island can be derived from a second quantization Hamiltonian as (see Methods)


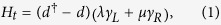


where the coupling coefficients *λ* and *μ* are assumed to be real and independent of *k* for simplicity. This Hamiltonian involves both the local coupling *λ* to an adjacent MBS at one end of the nanowire and the nonlocal coupling *μ* to the MBS at the other end of the nanowire (see Fig. 1). Due to its smaller magnitude, the nonlocal coupling was neglected in most previous studies^13^,^21^,^22^,^23^,^24^,^40^,^41^ with an exception of Ref. 21. We note that this nonlocal coupling can give rise to further detector-position-dependent measurement results which may also be used for the identification of the MBSs. The nonlocal coupling is therefore also considered here.

The coupling between two separated MBSs at the two ends of the nanowire can be described by^15^


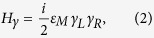


where 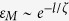 is the coupling energy with *l* being the wire length and ζ the superconducting coherent length. The pair of MBSs can constitute a regular fermion with operators


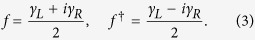


In this regular-fermion representation, the Hamiltonian 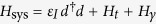 of the hybrid MBS-SET system becomes


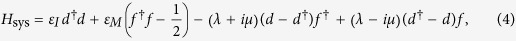


where *ε*_*I*_ is the resonant-level energy of the SET island and *d*^†^(*d*) is the corresponding creation (annihilation) operator. Note that this energy can be tuned by the gate voltage *V*_*g*_ to be zero (i.e., *ε*_*I*_ = 0) to ensure resonant tunnelings between the SET island and the zero-energy MBSs. The basis states of the system of interest are given by |*n*_*d*_*n*_*f*_〉, with *n*_*d*_ and *n*_*f*_ being 0 and 1, i.e., *a* ≡|00〉,*b* ≡|01〉,c ≡|10〉,*d* ≡|11〉. To compare the transport behaviors of the SET in the presence of the MBSs with those of a regular fermionic bound state in the nanowire, we also consider the following system Hamiltonian


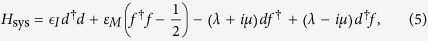


which describes the SET when coupled to a regular fermionic state.

The Hamiltonian for the source and the drain electrodes of the SET is described by


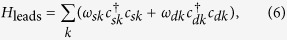


where *c*_*sk*_ (*c*_*dk*_) is the annihilation operator for electrons in the source (drain) electrode. The tunneling Hamiltonian between the SET island and the two electrodes is


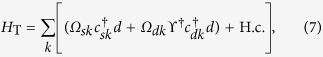


where Ω_*sk*(*dk*)_ is the coupling strength between the SET island and the source (drain) electrode. The counting operator ϒ (ϒ^†^) decreases (increases) the number of electrons that have tunneled into the drain electrode in order to keep track of the tunnelings of successive electrons. Thus, the total Hamiltonian of the system is given by *H*_tot_ = *H*_sys_ + *H*_leads_ + *H*_T_.

### Counting statistics

To study the FCS, it is essential to know the probability *P*(*n,t*) of 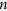 electrons having been transported from the SET island to the drain electrode during a period of time interval 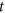. It is related to the cumulant generating function *G*(*χ*, *t*) defined by^46^


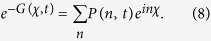


We will consider the time interval *t* much longer than the time for an electron to tunnel through the SET island (i.e., the zero-frequency limit), so that transient properties are insignificant. The derivatives of *G*(*χ*, *t*) with respect to the counting field *χ* at *χ* = 0 yield the cumulant of order 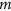 as


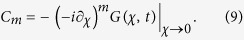


These cumulants carry complete information of the FCS on the SET island. For instance, the average current and the shot noise can be expressed as *I* = *eC*_1_/*t* and *S* = 2*e*^2^*C*_2_/*t*. Thus, the Fano factor *F* is given by *F* = *S*/2*eI* = *C*_2_/*C*_1_, which is used to characterize the bunching and anti-bunching phenomena in the transport process. The third-order cumulant *C*_3_ gives rise to the skewness *K* = *C*_3_/*C*_1_ of the distribution of transported electrons.

On the other hand, the probability distribution function of the transported electrons can be expressed as


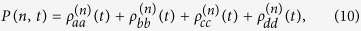


where 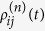 (*i*,*j* ∈ {*a*, *b*, *c*, *d*}) denote the reduced density matrix elements of the SET island at a given number 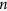 of electrons being transported from the SET island to the drain electrode at time *t*. We will calculate these reduced density matrix elements using a master equation (see Methods) which assume a large bias voltage across the SET. In fact, this large-bias case was considered in many previous studies^50^,^51^,^52^ as it is easy to implement in experiments. Moreover, this makes the problem simpler and more transparent because the broadening effect of the SET level can be neglected (see, e.g., Refs. 53 and 54). Using the discrete Fourier transform of the density matrix elements given by


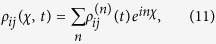


we can convert the master equation into


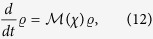
with


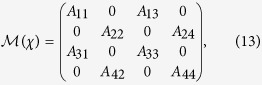


where 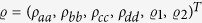 with 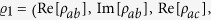

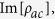

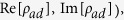
 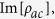
and 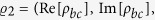 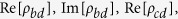 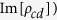, and


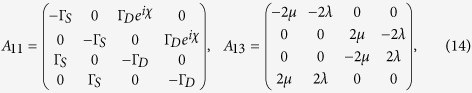



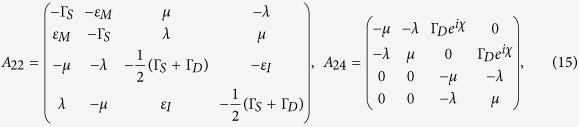



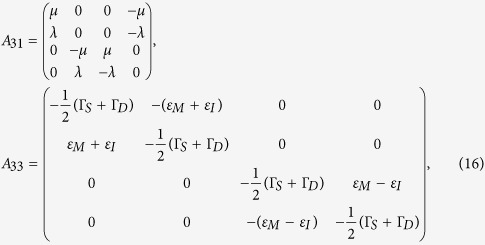



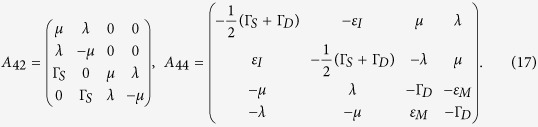


Here Γ_*S*(*D*)_ is the tunneling rate of the electrons through the barrier between the SET island and the source (drain) electrode and it is given by


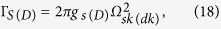


where *g*_*i*_ (*i* = *S*, or *D*) denotes the density of states at the source or drain electrode and is assumed to be constant over the relevant energy range.

The formal solution to the dynamical equation of 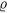 (*χ*, *t*) can be readily obtained as 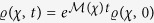. The cumulant generating function then reads *G*(*χ*, *t*) =  −lnT*rρ*(*χ*, *t*). At long time *t* (i.e., zero-frequency limit), the cumulant generating function is simplified to^55^


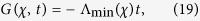


where Λ_min_(*χ*) is the minimal eigenvalue of 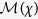 and satisfies Λ_min_|_*χ* → 0_ → 0 due to the probability normalization 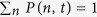.

### Signatures of the MBSs

**Figure 2:**
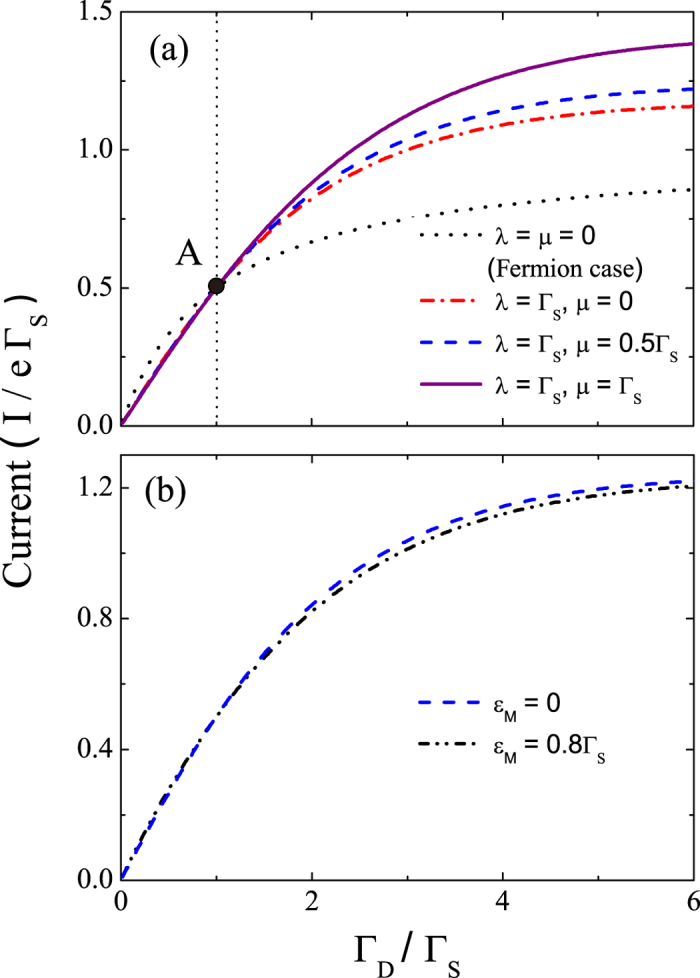
Current under the effect of the MBSs. Current *I* through the SET island to the drain electrode versus the tunneling-rate ratio Γ_*D*_/Γ_*S*_ for *ε*_*I*_ = 0. In (**a**), *ε*_*M*_ = 0 as *λ* and *μ* are varied. In (**b**), *λ* = Γ_*S*_, and *μ* = 0.5Γ_*S*_ as *ε*_*M*_ is varied.

(1). *Current.* Below we consider the zero-temperature case for the SET system since related experiments are usually performed at extremely low temperatures (see, e.g., Refs. 54 and 56). Figure. 2(a) shows the current flowing from the SET island to the drain electrode as a function of Γ_*D*_/Γ_*S*_ for *ε*_*M*_ = 0 and various values of *λ* and *μ*. In particular, the case of *λ* = *μ* = 0 represents the absence of the MBSs. Our calculation shows that it also equivalently represents the case of coupling to a fermion in the nanowire. This is expected because a regular fermion state does not affect the counting statistics of a nearby SET in the zero-frequency limit (or stationary behaviors) considered. It is clear from Fig. 2(a) that for a symmetric SET in which the tunneling rates between the SET island and the two electrodes are the same, i.e., Γ_*D*_ = Γ_*S*_, the current does not vary with *λ* and *μ* (see also the analytical result below). However, when Γ_*D*_ ≠ Γ_*S*_, the current in the presence of the MBSs deviates appreciably from that in the absence of the MBSs, especially in the region Γ_*D*_ > Γ_*S*_. Moreover, Fig. 2(b) shows that the current also changes, albeit slightly, when varying the coupling energy *ε*_*M*_ of the two MBSs . From Fig. 2(a,b), although coupling to the MBSs does change the current quantitatively, a distinct qualitative feature is lacking. Thus, it is insufficient to use only the current to show the existence of the MBSs.

Much of the above numerical results can also be obtained from analytic expressions in some special cases. For *ε*_*M*_ = 0, we obtain from equation (19) an analytical result for the current:


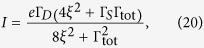


where Γ_tot_ = Γ_*S*_ + Γ_*D*_ and 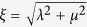. Although this symmetric property of the two couplings *λ* and *μ* has been noticed before^21^, we emphasize that we apply full counting statistics (including the Fano factor and the skewness as shown below) to reveal signatures of the MBSs, which goes beyond the conductance results reported in Ref. 21. When the MBSs are absent, i.e., *ξ* → 0, equation (20) recovers the well-known result


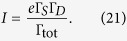


Alternatively, with the MBSs coupled and Γ_*S*_ = Γ_*D*_ = Γ, the current is reduced to *I* = *e*Γ/2, independent of the values of *λ* and *μ*.

When the MBSs are absent, the current through the SET island at zero temperature can also be calculated using^53^


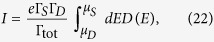


where *μ*_*S*(*D*)_ is the chemical potential of the source (drain) electrode, and *D*(*E*) is the density of states (DOS) of the SET island. When including the electrode-induced level broadening of the SET island, the broadened DOS can be described by a Lorentzian function^53^ centered around *E* = *ε*_*I*_:


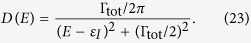


Therefore, the current can be calculated as
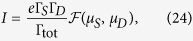
where
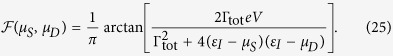


If the bias *eV* ≡ *μ*_*S*_ − *μ*_*D*_ applied on the SET is large so that the SET level *ε*_*I*_ is deeply inside the bias window, i.e., *eV* ∼ 2|*ε*_*I*_ − *μ*_*S*(*D*)_| ≫ Γ_tot_, the factor 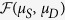 is simply reduced to


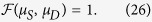


**Figure 3:**
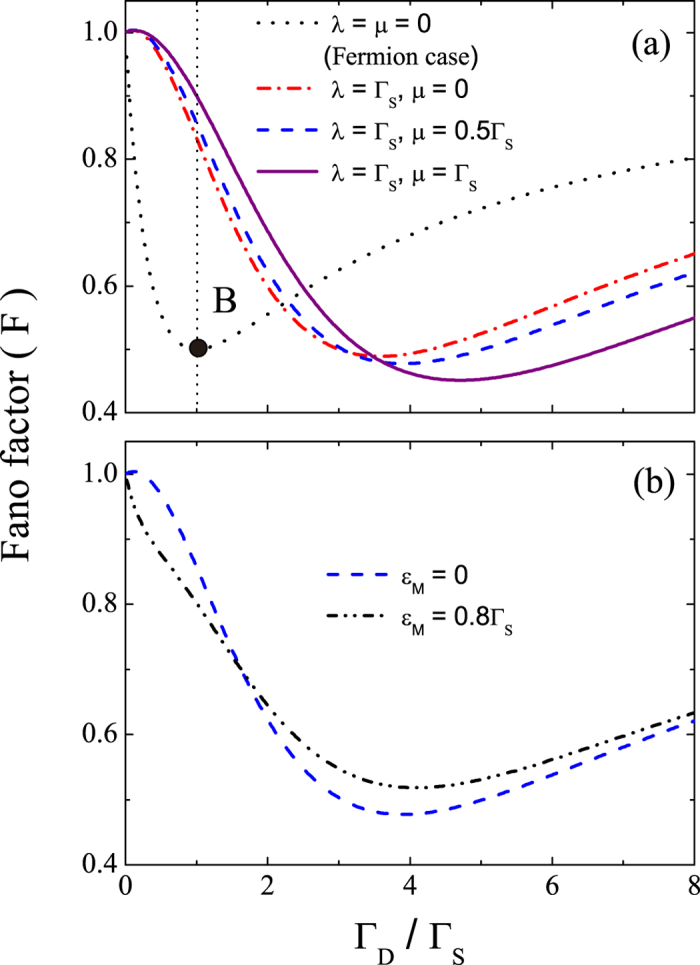
Fano factor under the effect of the MBSs. Fano factor *F* versus the tunneling-rate ratio Γ_*D*_/Γ_*S*_. The parameters in (**a**) are the same as those in Fig. 2(a), and the parameters in (**b**) are the same as those in Fig. 2(b).

Equation (24) then recovers^53^ equation (21).

(2). *Fano factor.* It is known that the current from the SET island to the drain electrode is related to the first-order cumulant of the generating function *G*(*χ*, *t*) by *I* = *eC*_1_/*t*. The corresponding shot noise is related to the second-order cumulant of *G*(*χ*, *t*) as *S* = 2*e*^2^*C*_2_/*t*. Thus, the Fano factor *F* = *S*/2*eI* can be written as *F* = *C*_2_/*C*_1_. In Fig. 3(a), we show the Fano factor as a function of Γ_*D*_/Γ_*S*_ for *ε*_*M*_ = 0 and various values of *λ* and *μ*. The black dotted curve in this figure represents the result not only for the case without the MBSs but also for the identical result for the fermion case similar to that in Fig. 1(a). It is clear that the Fano factor in the absence of the MBSs reaches its minimum (i.e., *F*_min_ = 1/2) for a symmetric SET with Γ_*D*_/Γ_*S*_ = 1, as indicated by point B on the black dotted curve in Fig. 3(a). This minimum point of the Fano factor shifts appreciably in the presence of the MBSs, e.g., *F*_min_ ≈ 0.49 at Γ_*D*_/Γ_*S*_ ≈ 3.58 when *λ* = Γ_*S*_ and *μ* = 0. Interestingly, this shift is robust against varying either the nonlocal coupling *μ* to the more distant MBS or the coupling energy *ε*_*M*_ between the two MBSs [see Fig. 3(a,b)].

For *ε*_*M*_ = 0, an analytical result for the Fano factor can also be obtained as


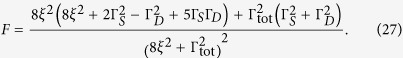


**Figure 4:**
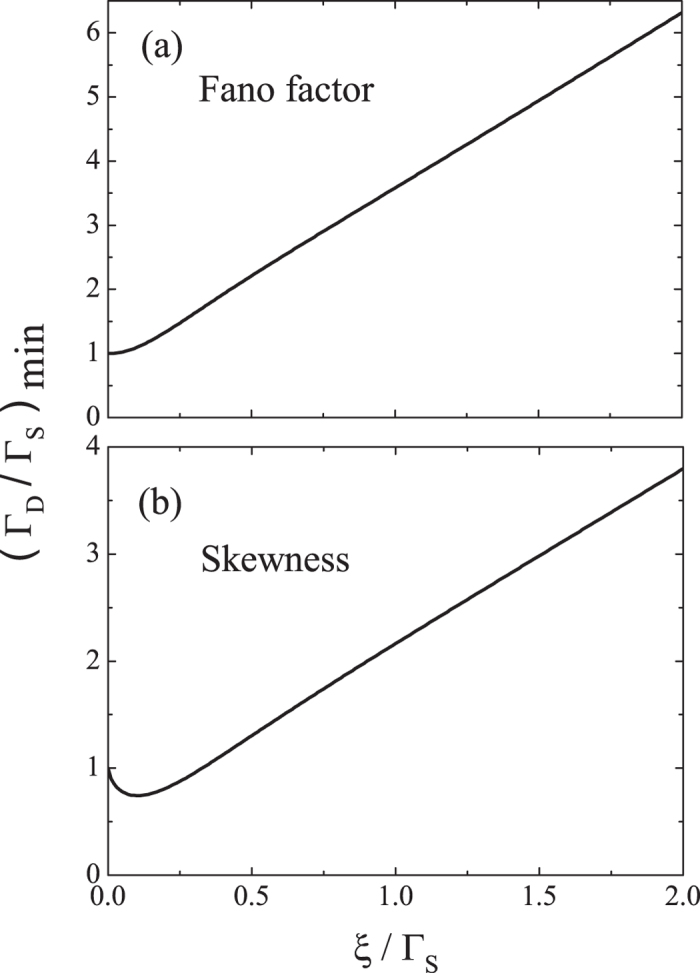
The minimum points of Fano factor and skewness under the effect of the MBSs. The minimum points (Γ_*D*_/Γ_*S*_)_min_ of (**a**) Fano factor and (**b**) skewness as a function of 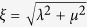.

**Figure 5:**
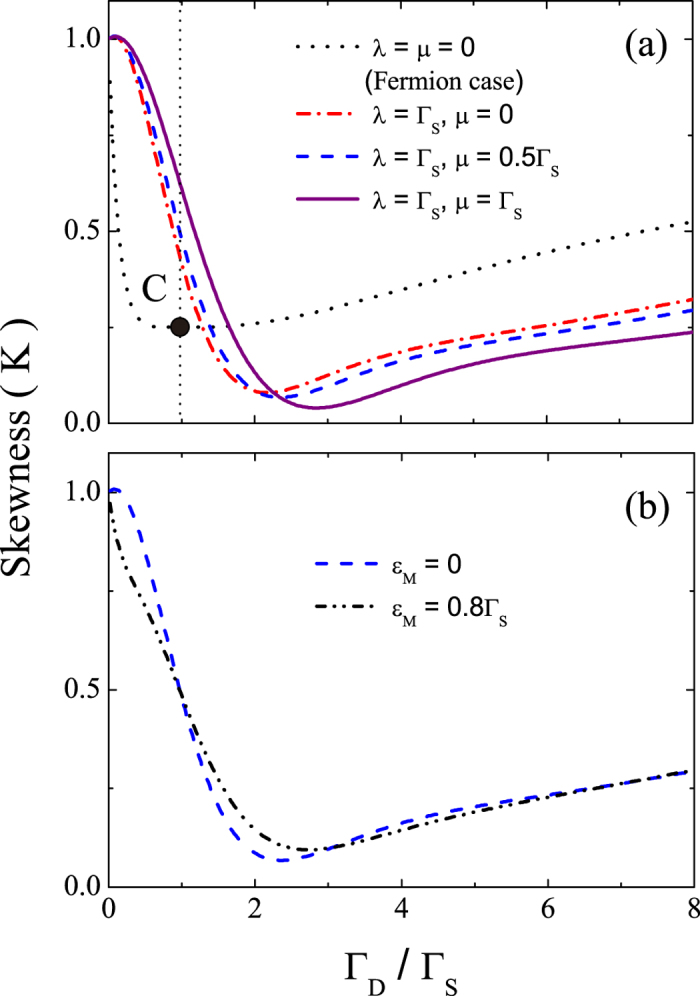
Skewness under the effect of the MBSs. Skewness *K* versus the tunneling-rate ratio Γ_*D*_/Γ_*S*_. The parameters in (**a**) are the same as in Fig. 2(a), and the parameters in (**b**) are the same as in Fig. 2(b).

Without the MBSs, i.e., *ξ* → 0, we recover the experimentally verified result^47^ *F* = (1 + *a*^2^)/2 < 1, where *a* = (Γ_*S*_ − Γ_*D*_)/Γ_tot_ is the asymmetry of the SET. In the presence of the MBSs and when Γ_*S*_ = Γ_*D*_ = Γ, the Fano factor follows *F* = (Γ^2^ + 4*ξ*^2^)/(2Γ^2^ + 4*ξ*^2^). It depends non-trivially on the couplings between the SET island and the MBSs, which does not occur for the current (see Fig. 2). In Fig. 4(a), we show the dependence of the minimum point from equation (27) on the SET-MBS coupling. We observe that the minimum point (Γ_*D*_/Γ_*S*_)_min_ of the Fano factor increases with *ξ*/Γ_*S*_. This MBS-induced shift of the minimum point of the Fano factor can be used as one of the signatures of the MBSs. Such a shift does not occur when coupled to a fermion state instead [see the black dotted curve in Fig. 3(a)]. We emphasize that we have considered a nanowire with both MBSs coupled to the quantum dot^21^,^42^. This generalizes results on the Fano factor from Ref. 40which considered coupling to only one MBS, which may not be applicable when the distances between the detector (e.g., SET) and two MBSs are comparable. In addition, instead of considering the Fano factors (or currents) at both the source and drain electrodes as in Ref. 40, we find it sufficient to characterize the MBSs by studying the statistics only at the drain electrode. This is in fact more directly related to typical experimental measurements. In particular, our results on the Fano factor (and also on the skewness as demonstrated below) can reduce back to the experimentally verified ones when the MBSs are decoupled as explained above. Moreover, tuning the gate voltages to control Γ_*D*_/Γ_*S*_ for identifying the MBSs in our proposal is a new alternative to the frequency tuning suggested in Ref. 40. Note that the shot noise of a quantum dot coupled to a MBS was explored in a more recent work^39^ to distinguish the Majorana origin of the zero-bias anomaly from that due to Kondo effect. However, their results of the shot-noise power spectra as well as the tunneling conductance were obtained under a smaller bias voltage (i.e., *eV*≪Γ_tot_). These are quite different from our results of Fano factor (or shot noise) and current which correspond to the case of a large bias voltage (i.e., *eV*≫Γ_tot_). In addition, we further explore the skewness below, which goes beyond the differential conductance (or current)^34^,^35^,^36^,^37^,^38^,^39^ and shot noise^39^ to reveal the signatures of MBSs.

(3). *Skewness.* The skewness of the distribution of transferred electrons is defined as *K* = *C*_3_/*C*_1_, which involves the third-order cumulant *C*_3_. Figure. 5(a) shows the skewness for *ε*_*M*_ = 0 and various values of 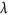 and 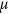. It is clear that the skewness in the absence of the MBSs takes its minimum value (i.e., *K* = 1/4) at Γ_*D*_/Γ_*S*_ = 1, as indicated by point C on the dotted curve. This dotted curve also represents the results for the fermion case due to the same reason as that for the result of the current or Fano factor as explained above. Also, the minimum point of the skewness shifts appreciably in the presence of MBSs, e.g., *K*_min_ ≈ 0.08 at Γ_*D*_/Γ_*S*_ ≈ 2.16 when *λ* = Γ_*S*_ and *μ* = 0. Moreover, similar to the Fano factor, this shift of the minimum point is also robust against varying *μ* and *ε*_*M*_ [see Fig. 5(a,b)].

If *ε*_*M*_ = 0, the skewness can be obtained analytically as


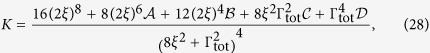


where


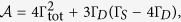



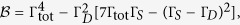



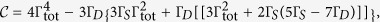



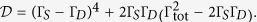


As expected, when *ξ* → 0 (i.e., the case with no MBSs), the skewness reduces to that of a SET: 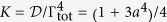, which was verified experimentally in Ref. 47. In the presence of the MBSs, the skewness takes its minimum value *K*_min_ at the minimum point (Γ_*D*_/Γ_*S*_)_min_ which can be accurately identified from equation (28) and is shown in Fig. 4(b). Similar to the Fano factor, this MBS-induced shift of the minimum point of the skewness can be used as another signature of the MBSs.

## Discussion

Note that the coupling strengths *λ* and *μ* of the SET island to the two MBSs at the ends of the nanowire depend on the position of the detector [see equation (34)]. Varying the position of the detector, one can reveal the influence of each MBS on the counting statistics (e.g., the Fano factor and the skewness) of the detector.

In our work, we use the Born-Markov master equation because it is applicable when both the couplings between the system and the electrodes are weak and each electrode has a wide flat energy spectrum. These conditions are valid in our system. Moreover, in studying the counting statistics of the SET island, we need to calculate the 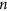-resolved reduced density matrix elements of the SET island [see equation (10)]. They are conveniently obtained using the master equation approach.

In summary, we have proposed an experimentally accessible approach to probe the MBSs via the counting statistics of a charge detector in the form of a SET. We study the effects of both the local coupling (to an adjacent MBS at one end of the nanowire) and the nonlocal coupling (to a MBS at the other end of the nanowire) on the counting statistics of the SET island. We find that in the presence of the MBSs, the minimum point of both the Fano factor and the skewness shifts appreciably from a symmetric SET configuration to an asymmetric one. This feature persists even when varying the nonlocal coupling to the farther MBS or the pairing energy between the two MBSs. These MBS-induced shifts can be used as signatures of the MBSs. Moreover, because our approach is based on the FCS, it can be readily generalized to higher-order cumulants to study if they can also be used to probe the MBSs.

## Methods

### Derivation of the tunneling Hamiltonian

For the two MBSs at the ends of a 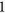
D *p*-wave superconductor, which can form at the interface between a semiconductor nanowire with strong spin-orbit coupling and an *s*-wave superconductor^14^,^18^, the Majorana operator can be defined as^18^,^41^


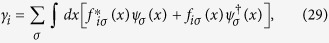


where *f*_*iσ*_(*x*), *i* = *L* or *R*, is the Majorana wave function and *ψ*_*σ*_(*x*) is the superconductor electron field operator with spin *σ* ( = ↑, ↓).

From equation (29), it follows that the Majorana operator satisfies 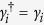. The anticommutation relation for the Majorana operators can be obtained as


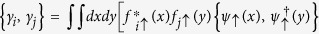



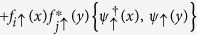



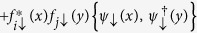



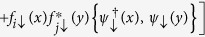



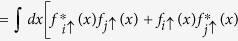



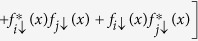



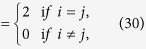


because of the anticommutation relations for the fermionic field operators
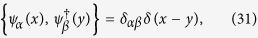

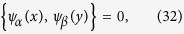


and the relations of the completeness and orthogonality of the Majorana wave functions


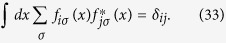


Obviously, it follows from equation (30) that 
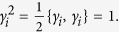


In the Nambu representation of the superconductor electron field operator, 
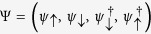
. Projecting these field operators onto the manifold of Majorana states, we have 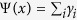 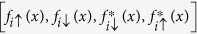. The electron tunnelings between the MBSs and the SET island are then described by the Hamiltonian


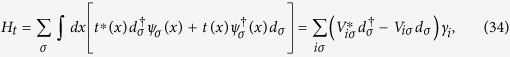


where 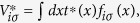 *d*_*σ*_ is the annihilation operator of the electron with spin 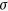 in the SET island, and 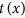 is the position-dependent coupling strength between the MBSs and the SET island. Note that one can always find suitable linear combinations of 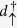 and 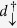 to form spinless fermions *d*^†^ coupled to the MBSs. Then, the tunneling Hamiltonian becomes


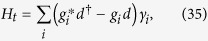


where operators *d*^†^ and *d* are defined as


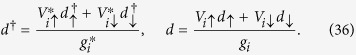


If 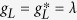 and 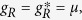 we have


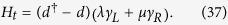


This is just the Hamiltonian in equation (1), which describes the electron tunnelings between the MBSs and the SET island. Note that it includes both the local coupling *λ* to the adjacent MBS at one end of the nanowire and the nonlocal coupling *μ* to the MBS at the other end of the nanowire. Equation (35) reduces to the tunneling Hamiltonian widely used in previous studies (e.g. Refs. 40 and 41) by choosing *μ* = 0.

### Quantum dynamics of the SET

Applying the Born-Markov approximation and tracing over the degrees of freedom of the electrodes coupled to the SET island, the master equation of the hybrid MBS-SET system in the Schrödinger picture can be obtained as


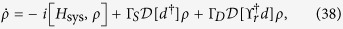


where *ρ*(*t*) is the reduced density operator of the MBS-SET system, and the superoperator 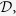 acting on any single operator, is defined as 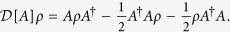


From equation (38) and the relations


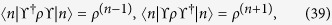



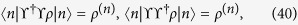


where *n* is the number of electrons that have tunneled to the drain electrode, we obtain the equation of motion for each *n*-resolved reduced density matrix element:


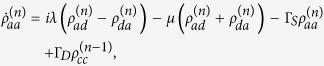



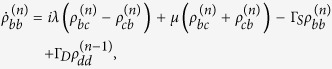



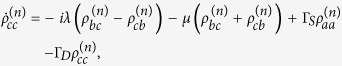



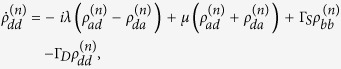



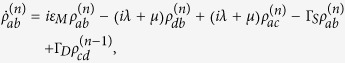



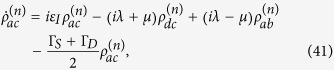



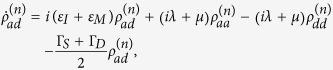



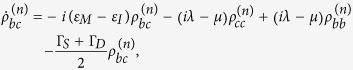



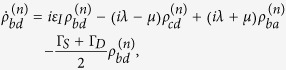



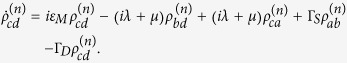


With the n-resolved matrix elements obtained, the reduced density matrix elements are given by 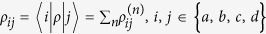.

## Additional Information

**How to cite this article**: Li, Z.-Z. *et al.* Probing Majorana bound states via counting statistics of a single electron transistor. *Sci. Rep.* **5**, 11416; doi: 10.1038/srep11416 (2015).
